# Anaphylaxis to Insect Venom Allergens: Role of Molecular Diagnostics

**DOI:** 10.1007/s11882-015-0527-z

**Published:** 2015-07-03

**Authors:** Markus Ollert, Simon Blank

**Affiliations:** 1 Department of Infection and Immunity, Luxembourg Institute of Health (LIH), 29, rue Henri Koch, L-4354 Esch-sur-Alzette, Luxembourg; 2 Department of Dermatology and Allergy Center, Odense Research Center for Anaphylaxis, University of Southern Denmark, Odense, Denmark; 3 Center of Allergy and Environment (ZAUM), Institute of Allergy Research, Technical University and Helmholtz Center Munich, Ingolstädter Landstraße 1, D-85764 Munich, Germany

**Keywords:** *Apis mellifera*, Component-resolved diagnosis, Hymenoptera venom, Insect venom allergy, Recombinant allergens, *Vespula vulgaris*

## Abstract

Anaphylaxis due to Hymenoptera stings is one of the most severe consequences of IgE-mediated hypersensitivity reactions. Although allergic reactions to Hymenoptera stings are often considered as a general model for the underlying principles of allergic disease, diagnostic tests are still hampered by a lack of specificity and venom immunotherapy by severe side effects and incomplete protection. In recent years, the knowledge about the molecular composition of Hymenoptera venoms has significantly increased and more and more recombinant venom allergens with advanced characteristics have become available for diagnostic measurement of specific IgE in venom-allergic patients. These recombinant venom allergens offer several promising possibilities for an improved diagnostic algorithm. Reviewed here are the current status, recent developments, and future perspectives of molecular diagnostics of venom allergy. Already to date, it is foreseeable that component-resolution already has now or will in the future have the potential to discriminate between clinically significant and irrelevant sensitization, to increase the specificity and sensitivity of diagnostics, to monitor immunotherapeutic intervention, and to contribute to the understanding of the immunological mechanisms elicited by insect venoms.

## Introduction

Allergy to the venom of Hymenoptera species is a classical IgE-mediated allergic disease caused by the crosslinking of receptor-bound IgE antibodies on the surface of mast cells and basophils. Hymenoptera venom allergy is one of the most severe hypersensitivity reactions with regard to the high risk of anaphylactic reactions with potentially fatal outcome.

Although venom allergy is one of the most frequent triggers of anaphylactic reactions in adults [1–3], the true number of fatalities may be underestimated since a study reports the presence of venom-specific IgE in 23 % of post-mortem serum samples taken from subjects, who had died outdoors suddenly and inexplicably between May and November [4]. Approximately 9.2 to 28.7 % of the adult population shows a sensitization to Hymenoptera venom [5], and the prevalence of systemic sting reactions among adults ranges between 0.3 and 7.5 % [5, 6]. A preferential association was observed between Hymenoptera venom allergy and mastocytosis [7•], since 20 to 39 % of patients with mastocytosis suffer from Hymenoptera venom allergy [5, 8, 9]. In addition to the higher prevalence of systemic reactions to Hymenoptera venom in adult patients with mast cell disorders, there are several reports which suggest that these patients are at risk for more severe reactions following stings [10–13].

Globally, all allergy-eliciting Hymenoptera belong to the suborder *Apocrita* which consists of the superfamilies *Apoidea* (*Apinae* and *Bombinae* subfamilies) and *Vespoidea* (*Vespinae*, *Polistinae*, *Formicinae*, and *Myrmicinae* subfamilies). In western and central Europe, the predominant elicitors of venom allergy are stings of honeybees (*Apis mellifera*) and yellow jackets (*Vespula vulgaris*). In southern Europe and the United States (US), additionally allergic reactions to paper wasps (*Polistinae*) are common [14]. In Europe, allergic reactions to ants are rare while they are of great importance in the US (especially *Solenopsis invicta*) [15] and Australia (especially *Myrmecia pilosula*) [16].

For patients with anaphylactic reactions to Hymenoptera venom, the only causative treatment which is effective in reducing the risk of subsequent systemic reactions is venom immunotherapy (VIT). Particularly in Hymenoptera, venom-allergic patients specific immunotherapy is very effective in inducing tolerance with a protection rate ranging from 75 to 98 % [17]. Prerequisite for the initiation of VIT should be the verification of an IgE-mediated reaction against the culprit venom. An unnecessary treatment with more than one or even with the wrong venom can lead to de novo sensitizations [18], increased risk of side effects and missing or limited protection to further stings and moreover, drastically increases the treatment costs.

The diagnosis of Hymenoptera venom allergy comprises the past medical history of a systemic sting reaction, a positive skin test response, and the detection of venom-specific IgE antibodies. Especially when the patient was not able to definitely identify the culprit insect, in clinical practice, the correct diagnosis is not always straightforward due to inherent problems and limitations of both tests. On the one hand, there are patients with a convincing history of anaphylaxis but negative diagnostic tests and, on the other hand, up to 50 % of patients show positive tests with more than one venom. Moreover, to date, no molecular tools are available which allow the prediction of the success of venom immunotherapy. Several limitations of diagnostic tests are based on the use of whole venom preparations for diagnosis. Venom preparations just like many other allergen extracts such as pollen or mite extracts [19, 20] often might show a highly variant allergen composition, which is essentially based on natural variability of the source material and additionally increased by different processing modalities or degradation of labile allergens, and in some cases, underrepresentation of particular allergens of high relevance. Hence, the reliability of diagnostic approaches is hampered by using venom extracts and the variant composition and low abundance of particular allergens even might affect therapeutic efficacy.

In recent years, significant progress has been made in the identification of novel Hymenoptera venom allergens, the detailed characterization of established allergens, and the development of suitable strategies for the recombinant production of venom allergens. To date, analyses on a molecular level are able to overcome at least some of the problems of diagnostic approaches and to contribute to an improved therapeutic intervention right now [21, 22••, 23, 24•].

## Hymenoptera Venom Allergens

Hymenoptera venoms are complex cocktails of low molecular weight substances such as biogenic amines, basic peptides, toxins, and of higher molecular weight proteins, many of them with enzymatic activity, together with a variety of other components, all of which may contribute to sensitization, allergic symptoms, and success of immunotherapy.

The certainly best characterized venom is that of the honeybee *Apis mellifera*, which surely is due to the outstanding importance of beekeeping and thus of the honeybee as elicitor of venom allergy all over the world and moreover, to the availability of detailed proteomic data of pure venom [25] and genomic information of the honeybee [26]. Additionally, in the last years, much progress has been made in the identification of new allergens of the yellow jacket (in Europe called common wasp) *Vespula vulgaris*. Among the best characterized honeybee venom (HBV), allergens are phospholipase A2 (Api m 1), hyaluronidase, (Api m 2) and melittin (Api m 4), all constituting medium to higher abundance proteins [27]. Prominent yellow jacket venom (YJV) allergens include phospholipase A1 (Ves v 1), hyaluronidase (Ves v 2.0101), and antigen 5 (Ves v 5), a protein of unknown function but high abundance in the venom [28, 29]. Recently, a second inactive hyaluronidase (Ves v 2.0201), carrying an inactivating mutation in the active site of the enzyme, was identified in YJV which interestingly seems to be the predominant isoform [30, 31].

Mainly by proteomic approaches in the last years, much progress has been made in identifying important allergens of low abundance. The gene of the well-known acid phosphatase (Api m 3) of HBV was identified and recombinantly produced [27, 32], and with the 100 kDa dipeptidyl peptidases IV (DPP IV) from HBV (Api m 5) and YJV (Ves v 3), a new class of homologous and cross-reactive Hymenoptera venom enzymes was identified [33]. Additionally, the 200 kDa vitellogenins Api m 12 and Ves v 6 were described as novel pair of cross-reactive panallergens of HBV and YJV [34]. Furthermore, it was demonstrated that Api m 10 (Icarapin, carbohydrate-rich protein) is a species-specific major allergen of HBV which might be of considerable interest for diagnostic as well as for therapeutic purposes [35••]. Very recently, it was demonstrated that at least nine additional Api m 10 transcript isoforms which are generated by alternative splicing or as intragenic chimeric transcripts are present in the venom gland [36]. The IgE reactivity with the Api m 10 isoforms, at least several of which are present in the venom proteome, is both, isoform- and patient-specific [36]. Other allergens of HBV include a putative protease inhibitor (Api m 6) [37, 38], a protease (Api m 7) [39], an esterase (Api m 8), a peptidase (Api m 9), and the two major royal jelly proteins (MRJP) 8 and 9 (Api m 11 isoforms) [40]. The role as major allergens to which more than 50 % of patients show IgE reactivity to so far was demonstrated for the HBV allergens Api m 1, Api m 2, Api m 3, Api m 5, and Api m 10 [41••]. Although, these five HBV allergens together with Api m 4 are able to detect IgE reactivity in approximately 95 % of patients with HBV allergy, the picture might be much more complex since at least 113 proteins and peptides were identified in HBV [42]. Moreover, the complexity is increased by different glycosylation patterns and protein heterogeneity [25, 36, 37, 43, 44] and even seasonal effects seem to influence the venom composition [45]. It can be anticipated that other Hymenoptera venoms will exhibit a comparable degree of complexity.

Bumblebee venom closely resembles honeybee venom and has two allergens of known sequence, phospholipase A2, and a protease. The honeybee and bumblebee venom phospholipases A2 show extensive sequence identity with each other, while no sequence identity is given with vespid phospholipase A1 [46], which differs in its specificity of the catalytic mechanism. The bumblebee has gained significantly in importance since it is increasingly used for pollination in greenhouses [47]. Similarly, the venoms of hornets, white-faced hornets, and paper wasps resemble YJV and contain phospholipases A1, hyaluronidases, and antigens 5, all of them exhibiting a high degree of sequence similarity. Moreover, fire ant venoms show high similarity with vespid venoms and contain a phospholipase A1 and an antigen 5. Varying from all other known Hymenoptera venoms, the major allergens of the *Myrmecia* venom are small peptides (pilosulins) which partially form homo- or heterodimers [48], but additionally, phospholipase and hyaluronidase activity was reported. A detailed overview about the presently known Hymenoptera venom allergens is given in Table 1.

**Table 1:** Overview of the hymenoptera venom allergens which are presently listed in the WHO/IUIS Allergen Nomenclature official database

Allergen	Name/Function	MW [kDa]	Potential N-glycosylation
**American paper wasps** (***Polistes annularis***, ***P*** *.* ***exclamans***, ***P*** *.* ***fuscatus***, ***P*** *.* ***metricus***)			
Pol a 1, Pol e 1	Phospholipase A1	34	0
Pol a 2	Hyaluronidase	38	2
Pol e 4	Protease	?	
Pol a 5, Pol e 5, Pol f 5, Pol m 5	Antigen 5	23	0
**Australian jumper ant** (***Myrmecia pilosula***)			
Myr p 1		7.5/5.5	0
Myr p 2	Pilosulin-3	8.5/2.4	0
Myr p 3	Pilosulin-4.1	4	0
Bees (***Apis mellifera***, ***A. cerana***, ***A. dorsata***)			
**Api m 1** ^a^, Api c 1, Api d 1	Phospholipase A2	16	1
**Api m 2**	Hyaluronidase	45	3
**Api m 3** ^a^	Acid phosphatase	49	2
**Api m 4** ^a^	Melittin	3	0
**Api m 5**	Allergen C/DPP IV	100	6
Api m 6	Protease inhibitor	8	0
Api m 7	Protease	39	3
Api m 8	Carboxylesterase	70	4
Api m 9	Carboxypeptidase	60	4
**Api m 10** ^a^	CRP/Icarapin	55	2
Api m 11.0101^a^	MRJP 8	65	6
Api m 11.0201^a^	MRJP 9	60	3
Api m 12	Vitellogenin	200	1
**Bumblebee** (***Bombus pennsylvanicus***, ***B. terrestris***)			
Bom p 1, Bom t 1	Phospholipase A2	16	1
Bom p 4, Bom t 4	Protease	27	0, 1
**European paper wasps** (***Polistes dominula***, ***P. gallicus***)			
Pol d 1, Pol g 1	Phospholipase A1	34	1
Pol d 4	Protease	33	6
**Pol d 5**, Pol g 5	Antigen 5	23	0
**Fire ants** (***Solenopsis invicta***, ***S. geminata***, ***S. richteri***, ***S. saevissima***)			
Sol i 1	Phospholipase A1	35	3
Sol i 2, Sol g 2, Sol r 2, Sol s 2		14	0
Sol i 3, Sol g 3, Sol r 3, Sol s 3	Antigen 5	26	2
Sol i 4, Sol g 4		12	0
**Hornets** (***Vespa crabro***, ***V. magnifica***, ***V. mandarinia***)			
Vesp c 1, Vesp m 1	Phospholipase A1	34	0
Vesp ma 2	Hyaluronidase	35	4
Vesp c 5, Vesp ma 5, Vesp m 5	Antigen 5	23	0
**Polybia wasp** (***Polybia paulista***, ***P. scutellaris***)			
Poly p 1	Phospholipase A1	34	0
Poly s 5	Antigen 5	23	0
**White-faced hornet, yellow hornet** (***Dolichovespula maculata***, ***D. arenaria***)			
Dol m 1	Phospholipase A1	34	2
Dol m 2	Hyaluronidase	42	2
Dol m 5, Dol a 5	Antigen 5	23	0
**Yellow jackets** (***Vespula vulgaris***, ***V. flavopilosa***, ***V. germanica***, ***V. maculifrons***, ***V. pensylvanica***, ***V. squamosa***, ***V. vidua***)			
**Ves v 1** ^a^, Ves m 1, Ves s 1	Phospholipase A1	35	0, 0, 2
Ves v 2.0101, Ves m 2	Hyaluronidase	45	4
Ves v 2.0201	Hyaluronidase (inactive)	45	2
Ves v 3	DPP IV	100	6
**Ves v 5** ^a^, Ves f 5, Ves g 5, Ves m 5, Ves p 5, Ves s 5, Ves vi 5	Antigen 5	25	0
Ves v 6	Vitellogenin	200	4

Allergens which are available for routine molecular diagnostics or most likely will become available in 2015 are printed bold

*CRP* carbohydrate-rich protein, *DPP IV* dipeptidyl peptidase IV, *MRJP* major royal jelly protein

^a^Marker allergens with experimental evidence to be able to discriminate each by itself between honeybee and yellow jacket venom allergy

## Recombinant Hymenoptera Allergens for Diagnosis

Only few allergens are present in substantial amounts in the venom. For honeybee venom, for example, Api m 1 and Api m 4 are predominant with amounts of dry weight of 12 and 50 %, respectively. Since low abundance allergens are difficult to isolate in substantial amounts, their recombinant availability can be considered a prerequisite for their detailed characterization and their use for diagnostic applications. However, even the purification of allergens of higher abundance from allergen extracts and their subsequent use for diagnosis has several disadvantages such as the danger of remaining impurities with other allergens and the presence of cross-reactive carbohydrate determinants (CCDs), both of which might severely impair and falsify analyses on a molecular diagnostic level.

All these problems can be bypassed by the application of recombinant technologies. Some allergens have been traditionally produced in bacteria, but for several venom allergens produced in this way, conformational IgE epitopes are affected due to the lack of proper posttranslational modifications and correct folding. Although, the bacterial system impresses with its production rates and cost-effectiveness, this recombinant approach is only feasible for structurally less complex allergens. So far, only the HBV allergen Api m 10 could be easily produced with authentic IgE reactivity in bacteria [35••]. Other small allergens produced in the prokaryotic system such as Api m 1 and Ves v 5 have to be subject to extensive refolding strategies to sustain comparable IgE reactivity to their native counterparts [49, 50]. For larger allergens, these strategies clearly will lead to limitations since for several allergens, posttranslational modifications such as the addition of glycan structures or correct disulfide bridging are imperative for correct folding and formation of conformational B cell epitopes. For such allergens, the recombinant production in eukaryotic cells from insect origin appears to be superior in terms of correct folding, glycosylation, and conservation of the full spectrum of epitopes [31, 33].

In the last decade, insect cell lines have developed to one of the most appropriate systems for the production of correctly folded authentic venom allergens [31, 33, 35••, 43, 51–53], which additionally offer considerable advantages compared to native allergens (see next paragraph). Moreover, some native allergens from Hymenoptera venoms such as Api m 1 were demonstrated to activate effector cells independent of IgE only by their enzymatic activity, thereby hampering cellular diagnostic assays [43, 54–56]. In contrast, recombinant strategies allow the inactivation of such activities without influencing the IgE reactivity of the allergen [43, 57].

So far, only the major allergens Api m 1 (phospholipase A2) of HBV, Ves v 1 (phospholipase A1) and Ves v 5 (antigen 5) of YJV, and Pol d 5 (antigen 5) of *Polistes dominula* venom are available for routine molecular diagnostics. Ves v 1 and Ves v 5 in different patient populations allow the identification of 92 to 96 % of patients with confirmed YJV allergy [58, 59, 60••]. In contrast, sensitivity of Api m 1 for the diagnosis of HBV allergy is lower and ranges from 58 to 80 % depending on the selection criteria of the patient population [41••, 61, 62•, 63, 64]. As a consequence of the diagnostic gap, which is created by using Api m 1 only for molecular IgE diagnostics, additional species-specific major allergens would be highly desirable. In a recent study, Api m 2, Api m 3, Api m 4, Api m 5, and Api m 10 in addition to Api m 1 were used in form of ImmunoCAP research prototypes to diagnose 144 patients with confirmed HBV allergy [41••]. The study demonstrated that not only Api m 1 but also Api m 2, Api m 3, Api m 5, and Api m 10 are major allergens to which more than 50 % of patients exhibit sIgE reactivity to, thus indicating that HBV contains a higher number of clinically important allergens than formerly anticipated. The combination of all 6 allergens showed a diagnostic sensitivity of approximately 95 % whereby 74 % of patients were sensitized to more than one allergen. Interestingly, the patient population showed 39 different sensitization profiles. Most of these allergens are currently being evaluated for clinical diagnostic use and will, once approved, provide important new diagnostic tools for clinicians managing patients with HBV anaphylaxis.

In addition to the added value of recombinant insect venom allergens in serological IgE diagnosis, also other diagnostic tests such as the basophil activation test could be improved by the additional use of recombinant venom allergens, thus leading to the development of more reliable and efficient in vitro tests for molecular allergy diagnostics [65].

## Molecular Diagnostics for the Dissection of Multiple Sensitizations

Positive results in skin testing or sIgE testing to conventional Hymenoptera venom extracts do not always reflect a clinically relevant sensitization [66]. In clinical practice, up to 50 % of patients show double-positive test results with honeybee and yellow jacket venom [67, 68]. In addition to true double sensitization to both venoms, these double-positive results are frequently caused by clinically irrelevant cross-reactive antibodies to cross-reacting carbohydrate residues. As many patients are not able to identify the culprit insect, a clinically relevant sensitization to both venoms cannot be excluded without further sIgE diagnosis on a molecular level. In the pre-molecular era, this has often led to an unnecessary treatment with both venoms resulting in higher costs, increased risk of side effects and possible de novo sensitizations [18].

**Fig. 1:**
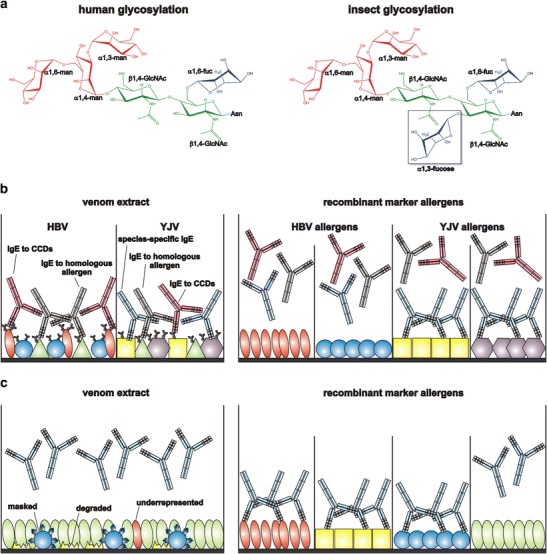
Molecular sIgE diagnostics: avoidance of glycan-specific binding and increased assay sensitivity. **a** Schematic representations of the core glycosylation of humans and insects. The latter carries an additional alpha-1,3-fucose residue which is not present in human carbohydrate structures and therefore is highly immunogenic. It can induce the generation of cross-reactive human IgE antibodies. (GlcNAc, N-acetylglucosamine; man, mannose; fuc, fucose). **b** Molecular diagnostics with recombinant marker allergens are able to exclude “false-positive” test results due to IgE directed against cross-reactive carbohydrate determinants (CCDs) or homologous allergens present in venom extracts. Thus, the detection of true sensitizations is markedly improved. **c** Molecular diagnostics with recombinant allergens is able to uncover IgE sensitizations to allergens that are underrepresented, labile, degraded, or masked in venom extracts and thereby helps to avoid “false-negative” test results

On the one hand, cross-reactivity may be based on the recognition of common protein epitopes of homologous allergens, present in both venoms as described for hyaluronidases (Api m 2 and Ves v 2), dipeptidyl peptidases (Api m 5 and Ves v 3) and vitellogenins (Api m 12 and Ves v 6) [33, 34]. On the other hand, the majority of cross-reactivities can be attributed to IgE antibodies that are directed against cross-reactive carbohydrate determinants (CCDs) (Fig. 1a, b) [69, 70]. This is of particular importance, since most Hymenoptera venom allergens are glycoproteins with one or more of such carbohydrate structures (Table 1). In insects, the relevant CCD epitope is defined by an alpha-1,3-linked fucose residue at the innermost N-acetylglucosamine of the carbohydrate core structure (Fig. 1a). Plants additionally carry a beta-1,2-xylose as second immunogenic modification. Since both glycan modifications are not present on human carbohydrate structures, they are highly immunogenic and can induce the production of specific IgG and IgE antibodies in humans [71]. IgE antibodies with specificity for the alpha-1,3-fucose epitope are responsible for approximately 75 % of double sensitizations to HBV and YJV [72]. The clinical relevance of these IgE antibodies appears to be rather low; however, they clearly affect diagnostic approaches since they cause multiple reactivities with any insect- or plant-derived glycoproteins. Thereby, CCD-specific IgE antibodies prevent the elucidation of clinically relevant sensitizations to protein epitopes and complicate the choice of the correct venom for immunotherapy (Fig. 1b). For the detection of the presence of CCD-specific IgE, nowadays, different reagents (ascorbate oxidase, bromelain, horseradish peroxidase, MUXF) have become available. However, since specific IgE directed against both, CCD and protein epitopes might be present, the detection of CCD-specific IgE does not allow the exclusion of sensitization to protein epitopes of multiple venoms [73]. So far, the only exceptions are the venoms of the paper wasps (*Polistinae*) which show no immunologically detectable CCD-reactivity [74].

Nowadays, molecular diagnostics using recombinant allergens has remarkable potential to discriminate between protein- and CCD-based IgE reactivity (Table 2). The use of *Spodoptera frugiperda* (Sf9) insect cells allows the recombinant production of properly glycosylated and thus correctly folded but CCD-free allergens due to a lack of immunologically detectable alpha-1,3-core fucosylation [31, 35••, 40, 43]. The advantage of CCD-free allergens is well illustrated by a comparison of native and recombinant CCD-free HBV major allergen Api m 1 [62•]. Both show a comparable diagnostic performance for CCD-negative patients. In contrast, for CCD-positive patients, native Api m 1 shows an increased reactivity only due to the CCD-reactivity of the native allergen, thereby demonstrating the superior performance of CCD-free allergens for CCD-positive patients. Moreover, the use of species-specific CCD-free allergens facilitates the exclusion of cross-reactivity due to protein epitopes of homologous allergens present in HBV and YJV.

**Table 2:** Examples of sIgE profiles in extract-based versus molecular diagnostics of honeybee and yellow jacket venom allergy, together with the deduced consequence for the decision on VIT based on the sIgE assay results

	sIgE of patients					
	1	2	3	4	5	6
Extract-based diagnostics						
HBV	+	+	+	−	+	−
YJV	+	+	+	−	+	−
Relevant venom	HBV/YJV	HBV/YJV	HBV/YJV	No	HBV/YJV	No
Molecular diagnostics						
Api m 1	+	+	−	−	−	−
Api m 3	+	−	−	+	+	−
Api m 4	−	−	−	−	+	−
Api m 10	+	−	−	+	+	−
Ves v 1	−	+	+	−	−	−
Ves v 5	−	+	+	−	−	+
CCD	+	+	+	−	+	−
Relevant venom	HBV	HBV/YJV	YJV	HBV	HBV	YJV

*CCD* cross-reactive carbohydrate determinant, *HBV* honeybee venom, *YJV* yellow jacket venom

Taken together, in most cases where the extract-based diagnostics does not allow the identification of the culprit venom due to clinically irrelevant cross-reactivity, the analysis on a molecular level applying species-specific venom allergens, devoid of CCDs, enables the detailed characterization of sensitization profiles and the identification of the venom causing clinical symptoms (Fig. 1b and Table 2) [59, 61, 64, 75, 76, 77••].

However, in the coming years, the field of molecular IgE diagnostics for the elucidation of multiple sensitization to Hymenoptera venoms will face additional challenges that need better solutions. In addition to their established importance in North America and Mediterranean regions of Europe, paper wasps, especially *Polistes dominula*, increasingly spread all over Europe as well as in the US from the warmer to the more moderate climate zones. Cross-reactivity between *Polistinae* and *Vespinae* (especially *Vespula* species) venoms is frequently observed [14, 78] independent of CCD-reactivity [74]. For *Polistes* venoms, only a very limited number of allergens is described, all of which are highly homologous to *Vespinae* allergens. Although it was proposed that *Polistes* and *Vespula* venom allergy should be discriminated by measurement of sIgE to antigens 5 (Pol d 5 and Ves v 5) and phospholipases (Pol d 1 and Ves v 1) [14], the known degree of sequence homology does not rule out extensive sIgE cross-reactivity based on protein epitopes. Thus, this approach only allows an estimation of the probably sensitizing venom. Moreover, the proposed discrimination relies on the amount of sIgE to the allergens of the different species. This is a factor which can depend on other causes than primary sensitization, such as quality of the allergen used for sIgE testing, and in many cases, results will be difficult to interpret. Thus, there is clearly a need for the identification and characterization of additional species-specific marker allergens for a more reliable diagnosis.

## Molecular Diagnostics in Patients With Undetectable Sensitization

Another diagnostic problem arises in patients with a convincing history of a systemic sting reaction but undetectable specific IgE in classical extract-based diagnostic approaches. A prominent example is a study comprising 308 patients with a systemic reaction to a yellow jacket sting [60••]. Only 83.4 % of the patients showed sIgE to the conventional YJV extract ImmunoCAP. In contrast, using the individual allergens Ves v 1 and Ves v 5, a sensitization was verified in 96 % of the patients. Interestingly, among the extract-negative patients, only one was tested positive for Ves v 1, whereas 84.4 % (42/51) showed a positive test with Ves v 5. Moreover, and in contrast to Ves v 1, in most extract-positive patients, the level of sIgE to Ves v 5 was substantially higher than the level to YJV extract, suggesting an underrepresentation of Ves v 5 epitopes in the extract. Applying a Ves v 5-spiked YJV ImmunoCAP, the sensitivity was increased from 83.4 to 96.8 % whereby this increase was not accompanied by a change in assay specificity. The Ves v 5-spiked YJV ImmunoCAP (i3) is now the commercially available standard product for one of the major allergy immunoassay suppliers [60••, 79]. In addition to the underrepresentation of Ves v 5 in the venom extract, other putative mechanisms for the reduced immunoreactivity could be an inefficient coupling of Ves v 5 to the solid phase of the assay or the masking of epitopes of the allergen by specific ligands which are present in the natural insect venom extract (Fig. 1c).

If a similar phenomenon also holds true for other venom allergens is not known so far. Furthermore, the example of the predominance of particular allergens such as Api m 1 and Api m 4 in HBV extract creates very likely a diagnostic information bias by favoring the detection of high abundant allergens in natural venom extracts. Recently, an underrepresentation or complete lack of the major allergens Api m 3 and Api m 10 was demonstrated for several therapeutic venom preparations which are licensed in European countries for routine venom immunotherapy [35••]. On the contrary, both allergens are detectable in crude venom. Thus, it is possible that downstream processing of the venom for immunotherapy medications affects the representation of major venom allergens, which can result in the loss of particular low abundance allergens with high clinical relevance. If this also is true for diagnostic products using venom extracts remains speculative at this point. However, most if not all of these aforementioned problems can be overcome by the use of an appropriate selection of recombinant venom allergens on molecular IgE diagnostic assay platforms (Fig. 1b, c).

Although controversially discussed [80–82], molecular diagnostics with other recombinant allergens than Ves v 5 seems to be useful for the improved detection of extract-negative patients [80, 81] depending on the quality of allergens used and the sensitivity of the assay platform [80, 83]. Additionally, we found that molecular diagnostics together with a diagnostic cut-off of 0.1 kU/L might be useful for the diagnosis of patients with low or undetectable sIgE to venom extract, especially for those with mastocytosis and/or elevated baseline serum tryptase, and can lead to a diagnostic sensitivity of 100 % for patients with YJV allergy (unpublished data).

## Molecular Diagnostics for the Prediction of Therapy Success

Apart from a well-documented field sting without a systemic reaction, the only recommended diagnostic method for the prediction of success of venom immunotherapy is the sting challenge with a living insect [84]. Most patients who are still reacting to a sting challenge while receiving conventional Hymenoptera venom immunotherapy will be protected by increased venom maintenance dosages [85, 86], whereby treatment failure rates are higher in patients suffering from bee venom allergy than from yellow jacket venom allergy [87•]. Since sting challenge tests can elicit severe systemic reactions, adequate in vitro methods for the prediction of success of immunotherapy would be more desirable. It appears possible that IgE analyses using molecular venom allergens might help to identify patients who are at risk to incompletely respond to conventional VIT. A possible consequence for those patients would be to initiate VIT already at a higher maintenance dosage from the very beginning and/or to apply specific companion diagnostics to them, such as sIgG4 measurements using recombinant venom allergens. As mentioned above the major HBV allergens Api m 3 and Api m 10, to which more than 50 % of HBV allergic patients exhibit significant IgE reactivity, are underrepresented or missing in several of the licensed HBV preparations routinely used for VIT [35••, 41••]. Of 144 patients with confirmed HBV allergy, 68 % showed sIgE reactivity with Api m 3 and/or Api m 10 and 4.8 % were sensitized to Api m 3 and/or Api m 10 exclusively [41••]. Moreover, in patients undergoing HBV VIT, a robust induction of allergen-specific IgG4 was observed to the highly abundant allergens Api m 1 and Api m 4, at a level comparable to with the specific IgG4 against whole venom extract. In contrast, a substantially lower induction of specific IgG4 to Api m 3 and Api m 10 was detected in the same study [41••]. So far, it remains speculative whether the lack of these particular allergens in certain therapeutic HBV preparations is a major factor contributing to the previously observed reduced efficacy of HBV VIT or whether other factors exist that still need to be identified. This hypothesis of the existence of potential sIgE sensitizations, which could be associated with a higher risk of therapeutic failure in HBV VIT, is currently under further clinical investigation by applying all the recent developments in the field of molecular sIgE diagnostics (personal communication Prof. P. Schmid-Grendelmeier, Zurich, Switzerland).

## Conclusions

Already today, molecular sIgE diagnostics of Hymenoptera venom allergy represent more than just an advanced diagnostic strategy. It has created added clinical value over the last decade. A component-resolution based on recombinant CCD-free species-specific allergens enables the differentiation between true sensitization and cross-reactivity and thus, in many patients improves the selection of the appropriate venom immunotherapy or the unnecessary therapy with multiple venoms instead of a single venom. Additionally, the availability of recombinant allergens facilitates to bypass the inherent limitations of venom extracts caused by heterogeneity and underrepresentation of particular important allergens. Moreover, for the future, component-resolved analyses possess the potential to improve the longitudinal monitoring of patients in the course of VIT on the level of sIgE and/or sIgG1/4 to molecular allergens. Further clinical studies will have to demonstrate, whether these new diagnostic tools will indeed provide superior diagnostic information to the clinician in charge of the venom-allergic patient, thus enabling the a priori identification of patients, at risk to inadequately respond to conventional extract-based venom immunotherapy.
